# Model guided extremum seeking control of electromagnetic micromirrors

**DOI:** 10.1038/s41598-021-97098-6

**Published:** 2021-09-02

**Authors:** Wanyan Sun, Yonghong Tan

**Affiliations:** 1 Tianjin Key Laboratory of Intelligent Robotics, and Institute of Robotics and Automatic Information System, College of Artificial Intelligence, Nankai University, Tianjin, 300350 China; 2 College of Information Mechanical and Electrical Engineering, Shanghai Normal University, Shanghai, 201418 China

**Keywords:** Information technology, Scientific data

## Abstract

In this paper, a simplified dynamic model is constructed to describe the main characteristic of electromagnetic micro-mirror. Then, based on the information provided by the derived simplified model, a model-guided extremum seeking control (MGESC) scheme with backtracking line search is developed, which can automatically estimate the best value of step-size at each search iteration to improve the performance of the control system for target tracking. Then, the convergence of the proposed MGES algorithm is proved. Finally, the experimental results and the simulations are presented to verify the proposed method.

## Introduction

**Figure 1:**
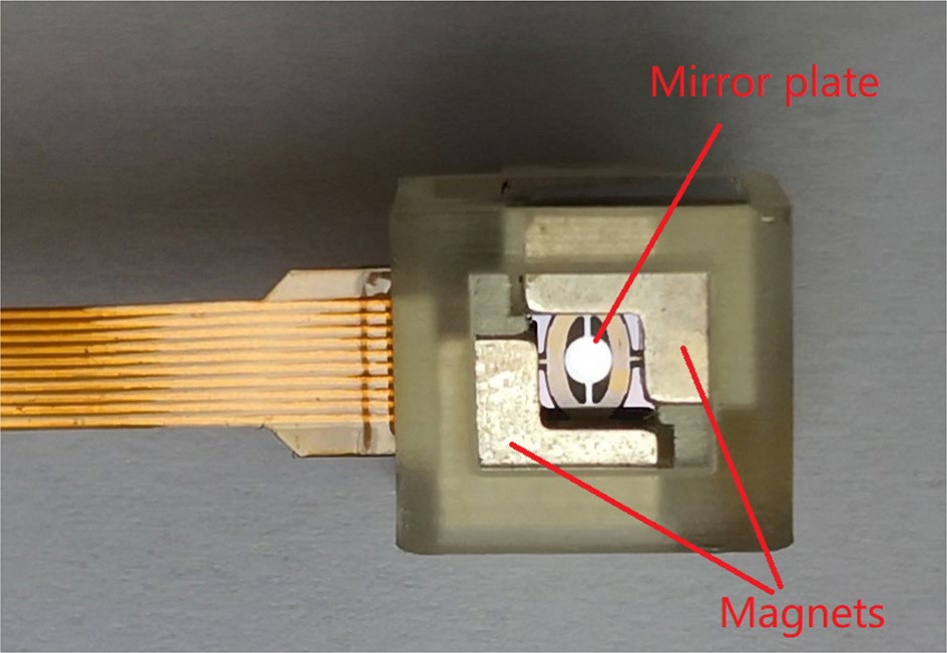
Electromagnetic micro-mirror.

Electromagnetic micromirror (EMM) as shown in Fig. 1 is a micro-electro-mechanical system (MEMS) device, which has the prominent advantage over other micromirrors in the aspects of lower power consumption, larger drive force, and larger deflection angle and so on^1–3^. Refs.^4,5^ presented the design and fabrication procedure of EMM as well as the piezoresistive sensor design for the measurement of deflection angle of EMM, but the open-loop control is used for angle control, which may lead to obvious tracking error especially when the working condition is changed. In order to obtain satisfactory tracking control performance, the optimization-based control strategy is one of the interesting options.

**Figure 2:**
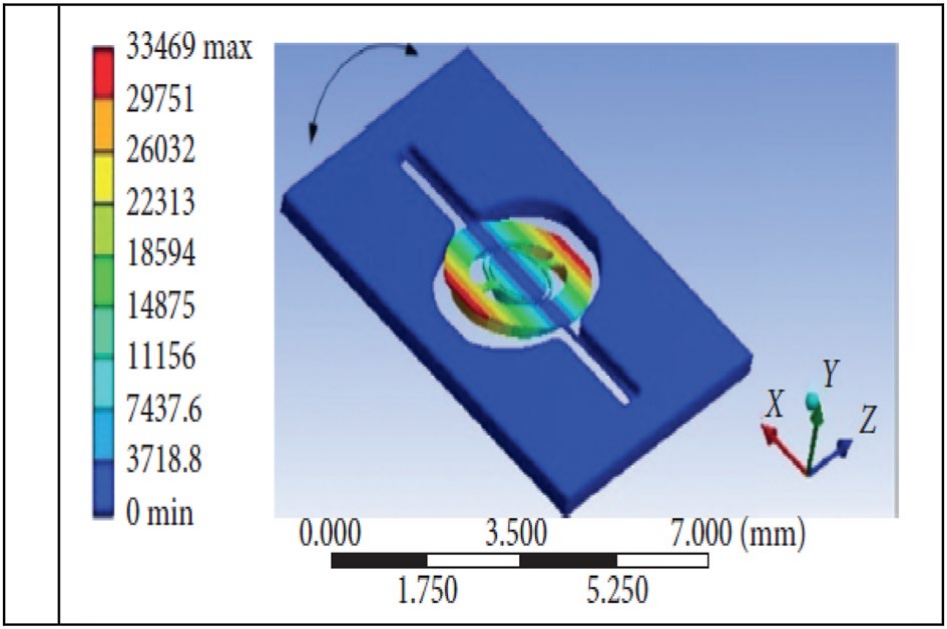
Main Resonant Movement in Slow-scanning Axis Control.

Ref.^6^ proposed a Newton’s optimization method to determine the harmonic coefficients of electromagnetic micromirror but the computational load is a significant challenge for engineering application. A method of Proportion-Integration-Differential (PID) control with low pass filter (LPF) was proposed in Ref.^7^. However, specifying the optimal gains for PID controller is not an easy task since the optimization procedure, in this case, is a nonconvex problem. In our previous work^8^, it is shown that the slow-scanning axis in the EMM system participated in several resonant motions under the signals of resonant frequencies, especially in the resonant movement on the slow-scanning axis shown in Fig. 2, which was the main reason of the instability in the control of electromagnetic micromirror. Therefore, the driving control of the slow scanning axis has reliability requirements compared to the harmonic driving control of the fast-scanning axis. Hence, the advanced control method needs to be explored for the slow-scanning axis control compared to the current existing control methods, in which the parameters are tuned usually by empirical techniques.

As we all know, for the systems that are difficult to build an accurate model, while maintaining the stability and boundedness of the signal, the so-called extreme seeking control (ESC) has the advantage of autonomously finding the optimal system behavior for target tracking^9,10^. Therefore, it will be chosen as the strategy for the slow-scanning axis control of EMM. Now, two problems are faced in exploring extremum seeking algorithm for slow-scanning axis control. One is that the first-principle model of the scanning axis is difficult to be constructed due to the complex structure and the complicated fabrication process of micromirror^5^, another is that the step-size of the ESC is tuned according to the empirical method^9^, which is not convenient for practical applications. Moreover, the selection of step-size based on empirical method cannot guarantee the best value of step-size is selected, which makes the ESC suffer from the slow convergence rate, which will degrade the control performance. Recently, Refs.^11,12^ proposed the concept of offline model-guided extremum seeking optimization for the optimal calibration of engine compression ignition and fuel injection optimization, which has the capability to accelerate convergence with the support of known models.

Inspired by the above-analysis, in this paper, a model-guided extremum seeking control (MGESC) strategy is proposed for angle control of EMM system. In this strategy a simplified linear model to describe the main characteristic of EMM system is constructed. To specify the model structure such as the order and coefficients of the model^13^, a cost function minimization-based method is developed. Furthermore, for the improvement of target-tracking performance of EMM, the model-guided extremum seeking algorithm with backtracking line search (BTLS) is proposed to search for the optimal control solution, and stabilize the system in the case that the resonance happens in the slow-scanning axis control. This method can automatically identify the best optimization step-size of iteration for extremum search. Finally, the simulation shows that the proposed algorithm has achieve promising result.

## Modeling of EMM

### Schematic of electromagnetic micromirror system

**Figure 3:**
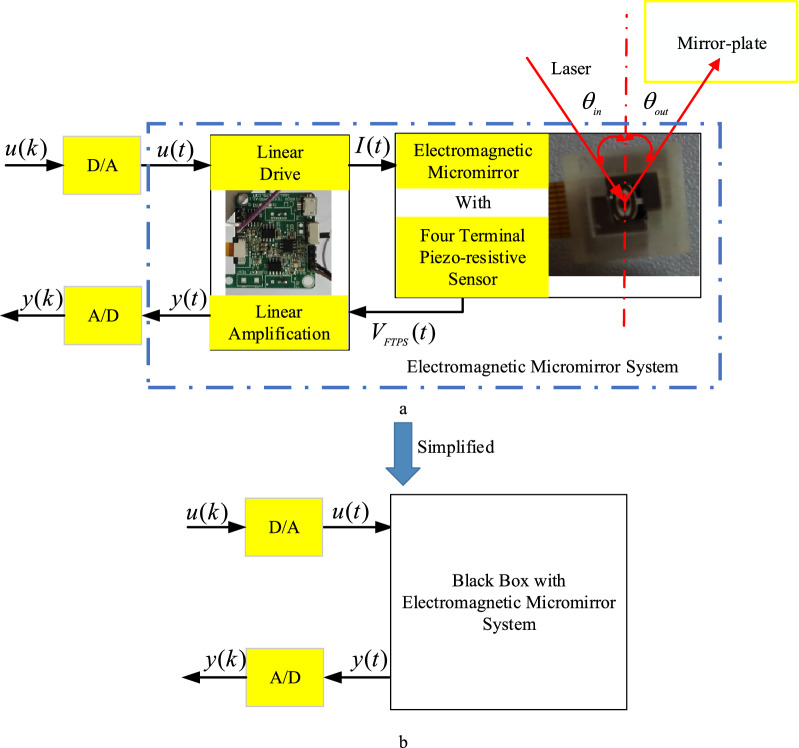
Schematic of Electromagnetic Micromirror System.

Schematic of electromagnetic micromirror system shown in Fig. 3a. In the EMM, the slow-scanning axis control, $u(t)$ is the input voltage and $y(t)$ is the output of the piezoresistive angle sensor used to measure the angle position of the slow-scanning axis in the EMM, respectively. The study in this paper just focuses on the angle control of slow-scanning axis. The output of control algorithm generated by the computer is sent out via D/A converter to produce driving electric current $I(t)$ to drive the slow-scanning axis of the EMM. The piezo-resistive sensor (PRS) is used to measure the angle of EMM. The corresponding output voltage of the PRS, i.e., V_FTPS_ is amplified by the linear amplifier to produce the output voltage $y(t)$, which is then sampled by the A/D converter to feedback to the computer. In the method of this paper, the behavior of the slow-scanning axis of EMM is considered as a black box as shown in Fig. 3b. Based on the structure shown in Fig. 3b, using the measured input and output of the EMM, the simplified model is established.

### Establishment of simplified model

It is known that the deflection mechanism of the EMM can be described by

1 $$ M\ddot{y} + B\dot{y} + Ky = T_{e} , $$

where *y* is the deflection angle, *M* is the mass moment of inertia, *B* is the damping coefficient, *K* is the spring constant of flexure, and *T*_*e*_ is the electromagnetic torque which is described as^8^

2 $$ T_{e} = H[\Phi ,u] = \frac{\Phi }{R}\sum\limits_{i = 1}^{n} {\oint\limits_{{L_{i} }} {r_{i} } } dl_{i} u\sin \theta $$

where *Ф* is the external magnetic field generated by the permanent magnets, *u* is the control voltage and *R* is the resistance of the spial coil, *r*_*i*_ is the distance between the unit length of the coil and the axis of rotation on the *i*th coil, and $\theta$ is the angle between $\vec{I}$ and $\vec{B}$. From (2) and the detailed analysis of torque to drive the mirror plate^14^, it is known that the relation between the control voltage and the drive torque has a complex mapping. However, based on the model described by formulas (1) and (2), it is not convenient to design a suitable controller to control the angle of the micromirror.

By ignoring the nonlinear factor, such as hysteresis^15,16^ in the EMM, a simplified model is used to describe the major dynamic behavior of the EMM. The purpose of building the simplified model is to use it to provide the initial search direction to ESC mechanism to speed-up the convergence and to obtain good control performance. Suppose the system can be described as

3 $$ y(k) = \hat{y}(k) + e(k), $$

with the simplified model

4 $$ \hat{y}(k) = \psi^{T} (k)\hat{\theta }(k), $$

where the parameter vector is

5 $$ \hat{\theta }^{T} (k) = \left[ {\begin{array}{*{20}c} {\hat{a}_{1}^{k} } & {\hat{a}_{2}^{k} } & \cdots & {\hat{a}_{m}^{k} } & | & {\hat{b}_{1}^{k} } & {\hat{b}_{2}^{k} } & \cdots & {\hat{b}_{m}^{k} } \\ \end{array} } \right]_{1 \times 2m} $$

and data vector is

6 $$ \psi^{T} (k) = \left[ {\begin{array}{*{20}c} { - y(k - 1)} & { - y(k - 2)} & \cdots & { - y(k - m)} & | & {u(k - 1)} & {u(k - 2)} & \cdots & {u(k - m)} \\ \end{array} } \right]_{1 \times 2m} $$

where $u(k)$ and $y(k)$ are discrete-time input voltage and output voltage sampled at time step $k$ respectively; $m$ is the model order. Define

7 $$ e(k) = y(k) - \hat{y}(k) $$

as the modeling error which is supposed to be a white noise with zero-mean value and variance σ^2^. Then, the following formula

8 $$ V(m,N) = E^{T} (k)E(k) = \sum\limits_{k = 1}^{N} {e^{2} (k)} , $$

is defined as the cost-function to evaluate the modeling performance, where $N$ is sample number, and

9 $$ E^{T} (k) = \left[ {\begin{array}{*{20}c} {e(1)} & {e(2)} & \cdots & {e(k)} \\ \end{array} } \right]_{1 \times k} $$

is the error vector. It is known that the order of the model is to determine the structure of the model. Considering the incremental variation of the cost function, i.e.,

10 $$ \Delta V(\hat{m},N) = V(\hat{m},N) - V(\hat{m} + 1,N) $$

the model order, $\hat{m}$, can be determined by checking whether

11 $$ \Delta V(\hat{m} + 1,N) \ll \Delta V(\hat{m},N). $$

is held, which means that no significant improvement of the cost function can be achieved, if so, then it adopts

12 $$ m = \hat{m}. $$

Because the simplified model is a linearized model and the *e*(*k*) is assumed to be the white noise with zero-mean value, the recursive least square (RLS) algorithm^13^ can be used to estimate the parameters of the model, which has simple structure and leads to unbians estimation of the environment with white noise. The corresponding algorithm is as follows:

13 $$ \hat{\theta }(k) = \hat{\theta }(k - 1) + K(k)(y(k) - \psi^{T} \hat{\theta }(k - 1)), $$

14 $$ K(k) = P(k - 1)\psi (k)(\psi^{T} (k)P(k - 1)\psi (k) + 1)^{ - 1} , $$

15 $$ P(k) = (I - K(k)\psi^{T} (k))P(k - 1). $$

where $K(k)$ is the gain matrix, $P(k)$ is the covariance matrix and $\hat{\theta }(k)$ is the estimation of the parameter vector.

## Model guided extremum seeking control scheme

Based on the obtained simplified model:

16 $$ A(z^{ - 1} )y_{{}}^{i} (k) = B(z^{ - 1} )x_{{}}^{i} (k), $$

where

17 $$ A(z^{ - 1} ) = 1 + a{}_{s,1}z^{ - 1} + \cdots + a_{s,m} z^{ - m} $$

and

18 $$ B(z^{ - 1} ) = b_{s,1} z^{ - 1} + \cdots + b_{s,m} z^{ - m} , $$

the corresponding discrete-time state space model can be derived by

19 $$ X_{s}^{i} (k + 1) = F_{s} X_{s}^{i} (k) + G_{s} u_{s}^{i} (k), $$

20 $$ y_{s}^{i} (k) = C_{s} X_{s}^{i} (k), $$

where

21 $$ X_{s}^{i} (k + 1) = \left[ {\begin{array}{*{20}c} {x_{s,1}^{i} (k + 1)} \\ {x_{s,2}^{i} (k + 1)} \\ \cdots \\ {x_{s,m - 1}^{i} (k + 1)} \\ {x_{s,m}^{i} (k + 1)} \\ \end{array} } \right] $$

is the state vector, $u_{s}^{i} (k)$ is the control voltage $y_{s}^{i} (k + 1)$ is the output of EMM,* i* is the iteration index and *k* is the sampling step. Moreover, in (19),

22 $$ F_{s} = \left[ {\begin{array}{*{20}c} 0 & 1 & 0 & \cdots & 0 \\ 0 & 0 & 1 & \cdots & 0 \\ \vdots & \vdots & \vdots & \cdots & \vdots \\ 0 & 0 & 0 & \cdots & 1 \\ { - a_{s,m} } & { - a_{s,m - 1} } & { - a_{s,m - 2} } & \cdots & { - a_{s,1} } \\ \end{array} } \right]_{m \times m} $$

is the state transfer matrix,

23 $$ G_{s} = [h_{s,1} \, h_{s,1} \cdots \, h_{s,m} ]^{T} $$

is the input matrix and

24 $$ C_{s} = [\begin{array}{*{20}c} 1 & 0 & \cdots & 0 \\ \end{array} ] $$

is the output vector. In addition, the parameters $$\begin{array}{*{20}c} {h_{s,1} ,} & {h_{s,2} ,} & {h_{s,3} ,} & { \cdots ,} & {h_{s,m} } \\ \end{array}$$ in the input matrix can be expressed as

25 $$ \begin{array}{*{20}c} {h_{s,1} = b_{s,1} } \\ {h_{s,2} = b_{s,2} - a_{s,1} h_{s,1} } \\ {h_{s,3} = b_{s,3} - a_{s,2} h_{s,1} - a_{s,1} h_{s,2} } \\ \vdots \\ {h_{s,m} = b_{s,m} - a_{s,m - 1} h_{s,1} - \cdots - a_{s,1} h_{s,m - 1} .} \\ \end{array} $$

In order to develop the model-guided extremum seeking algorithm with backtracking line search, define

26 $$ J_{s}^{i} (k + 1) = \frac{1}{2}[e_{s}^{i} (k + 1)]^{2} = \frac{1}{2}[r_{s} (k + 1) - y_{s}^{i} (k + 1)]^{2} $$

as the cost function of extremum seeking control at the $ith$ iteration of step $k + 1$, where $r_{s} (k + 1)$ is the target trajectory at step time $k + 1$, $u_{s}^{i + 1} (k)$ is obtained based on the steepest descent method at step $k$ for the $(i + 1)th$ iteration, i.e.,

27 $$ u_{s}^{i + 1} (k) = u_{s}^{i} (k) + \Delta u_{s}^{i} (k), $$

where

28 $$ \Delta u_{s}^{i} (k) = - \lambda_{s}^{k} \nabla J_{s}^{i} (k + 1) $$

and

29 $$ \begin{aligned} \nabla J_{s}^{i} (k + 1) &= \frac{{\partial J_{s}^{i} (k + 1)}}{{\partial u_{s}^{i} (k)}} \hfill \\ & = - (r_{s} (k + 1) - y_{s}^{i} (k + 1))\frac{{\partial y_{s}^{i} (k + 1)}}{{\partial {}_{s}x_{1} (k + 1)}}\frac{{\partial {}_{s}x_{1} (k + 1)}}{{\partial u_{s}^{i} (k)}} \hfill \\ \end{aligned} $$

where $\nabla J_{s}^{i} (k + 1)$ is the gradient of the cost function with respect to the input at the $ith$ iteration of step $k + 1$. From (29), it is noted that the gradients $\frac{{\partial y_{s}^{i} (k + 1)}}{{\partial {}_{s}x_{1} (k + 1)}}\frac{{\partial {}_{s}x_{1} (k + 1)}}{{\partial u_{s}^{i} (k)}}$ can be estimated based on the state-space model shown in (19) and (20). Therefore, different from the traditional ESC scheme without relying on any system models, the MGESC scheme uses the information provided by the simplified model as the start of iteration to search for the optimal or at least the sub-optimal solution of the control within each sampling step.

**Figure 4:**
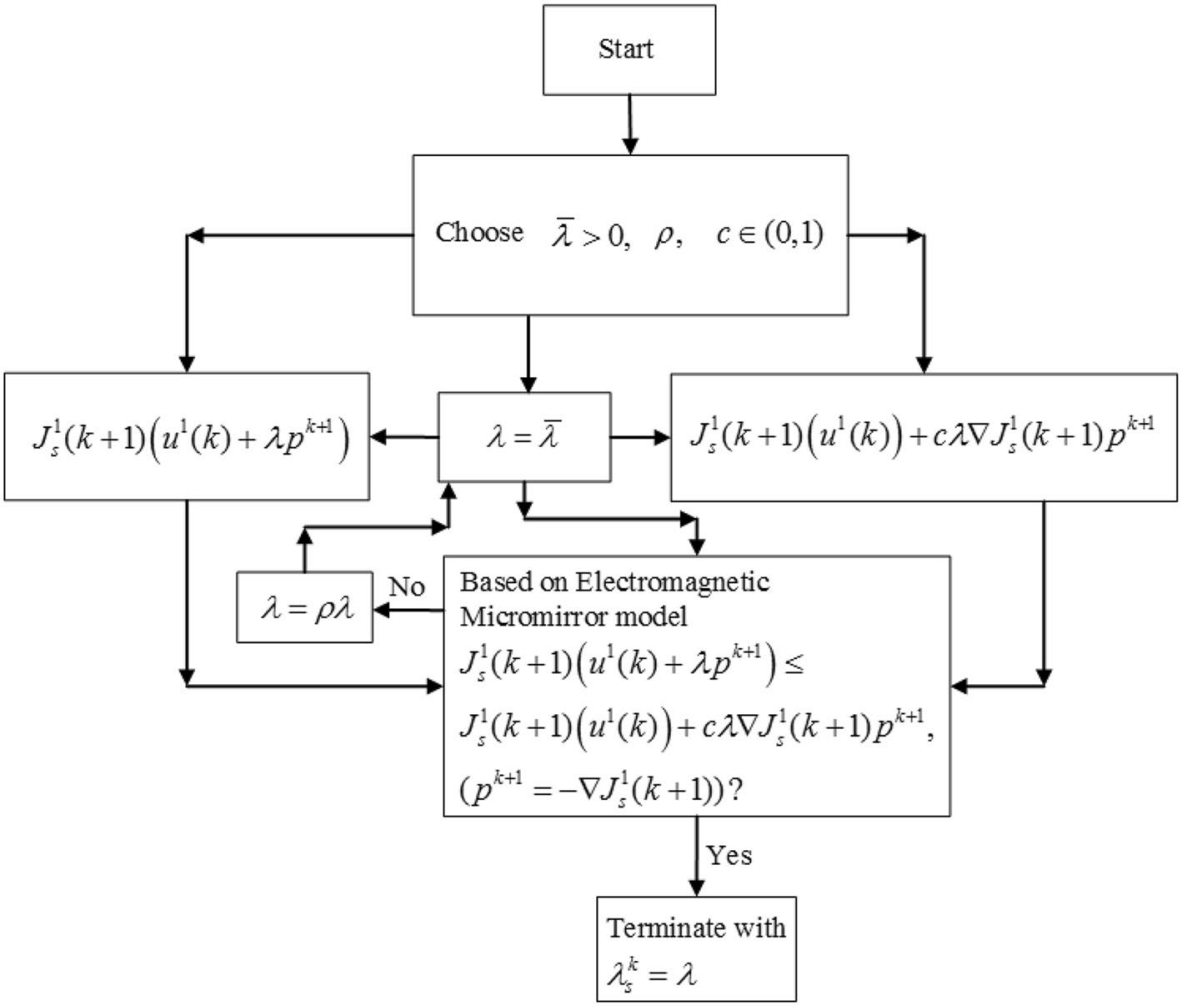
Flowchart of backtracking line search.

Moreover, the convergent performance of the MGESC is dependent on the appropriate selection of step size $\lambda_{s}^{k}$ at each step. In order to obtain the optimal step-size of extremum seeking process within each sampling step, the backtracking line search (BTLS) technique^17^ is applied to choosing the best step size $\lambda_{s}^{k}$ from the candidates of step size, and the corresponding flowchart of backtracking procedure is shown in Fig. 4.

From Fig. 4, the algorithm of BTLS can be concluded as.

1. Choose the initial values of $\overline{\lambda } > 0,$ and $\rho ,c \in (0,1).$
2. Let $\lambda = \overline{\lambda }$ and repeat $\lambda = \rho \lambda ,$ until
  30 $$ J_{s}^{1} (k + 1)(u^{1} (k) + \lambda p^{k + 1} ) \le J_{s}^{1} (k + 1)(u^{1} (k)) + c\lambda \nabla J_{s}^{1} (k + 1)p^{k + 1} $$
  where $$p^{k + 1} = - \nabla J_{s}^{1} (k + 1)$$.
3. In the end, let $\lambda_{s}^{k} = \lambda .$

With the BTLS algorithm, the extremum seeking algorithm is more flexible in search for the best optimization step size from the candidates of step sizes, which is better than the extremum seeking algorithm with fixed step size chosen via empirical method. By using the BTLS approach, the corresponding MGESC with BTLS algorithm is obtained by repeating the implementation of (19), (20), and (26)–(29) at each step, until the stop criterion

31 $$ J_{s}^{i} (k + 1) \le \varepsilon , $$

where $\varepsilon$ is supposed to be $10^{ - 8}$, is satisfied. Thus, it leads to

32 $$ u_{s}^{i} (k + 1) = u_{s}^{i + 1} (k). $$

**Figure 5:**
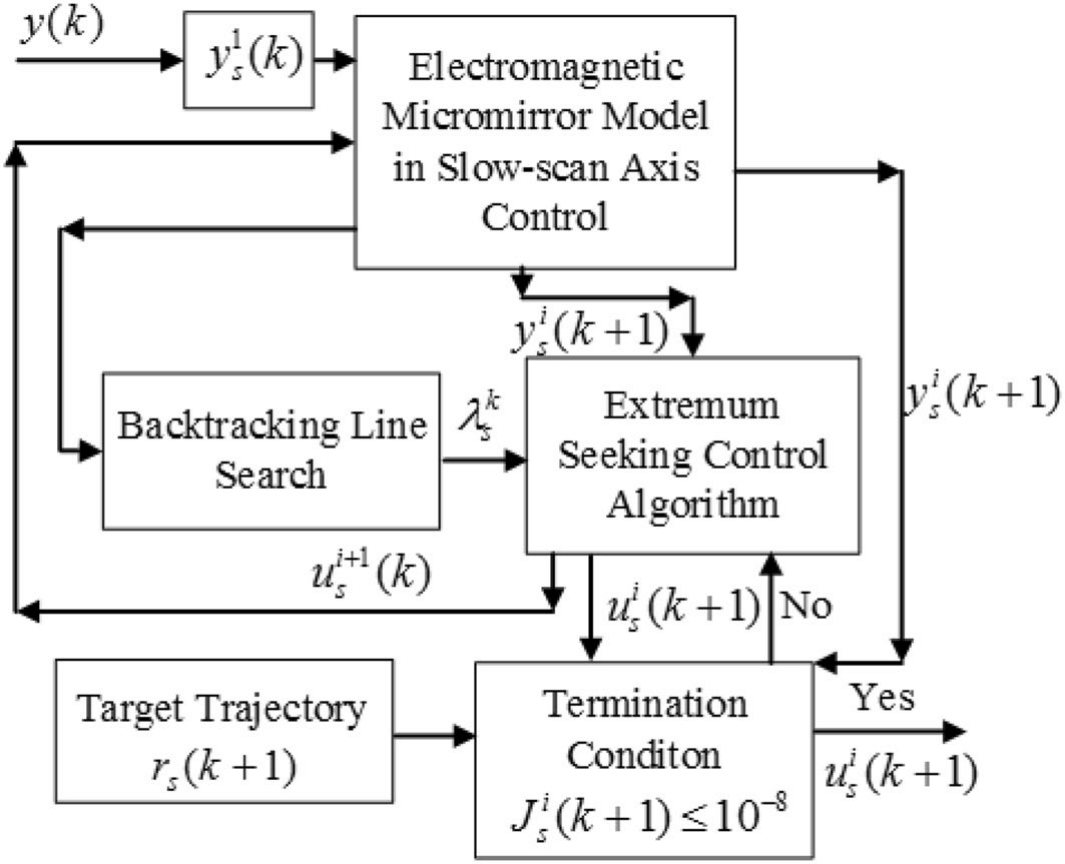
Flowchart of MGESC with BTLS algorithm.

Figure 5 illustrates the corresponding flowchart of the MGESC with BTLS algorithm.

Note that $x_{s,1}^{i} (k + 1)$ and $x_{s,2}^{i} (k + 1)$ are assigned to $x_{s,1}^{i + 1} (k)$ and $x_{s,2}^{i + 1} (k)$, respectively after the calculation of the simplified model shown as (19) and (20). In addition, in the MGESC with BTLS algorithm, the angle position, $y(k)$, is assigned to $y_{s}^{1} (k)$ as the starting value of the iteration in the extremum seeking mechanism. It is calculated to obtain the prediction of the output, i.e., $y_{s}^{i} (k + 1)$. Then substitute $y_{s}^{i} (k + 1)$ in $J_{s}^{i} (k + 1)$ and check whether stop criterion shown as (31) is satisfied. If so, the optimal solution of control is achieved, otherwise the extremum seeking control based on the obtained model continues the itserative calculation. The BTLS method chooses the best optimization step size $\lambda_{s}^{k}$ from the candidate step sizes for each extremum search at sampling step $k$.

### *Remark 1*

 From Eqs. (27)–(29), it can be seen that the performance of the ESC control scheme depends on the optimization step-size as well as the accurate estimation of the gradient. If the estimation of gradient is not correct, it may lead to poor control performance. Therefore, the MGESC method proposed in this paper uses the information provided by the simplified model to start the optimal search to obtain the accurate gradient estimation. On the other hand, the best-selection of step-size also has an important impact on the performance of the control system. Therefore, the BTLS method used in this paper can seek the best result from the candidates of the optimization step-size. The combination of these two aspects can ensure that the satisfactory result can be obtained for the ESC system.

## Modeling and simulation results

**Figure 6:**
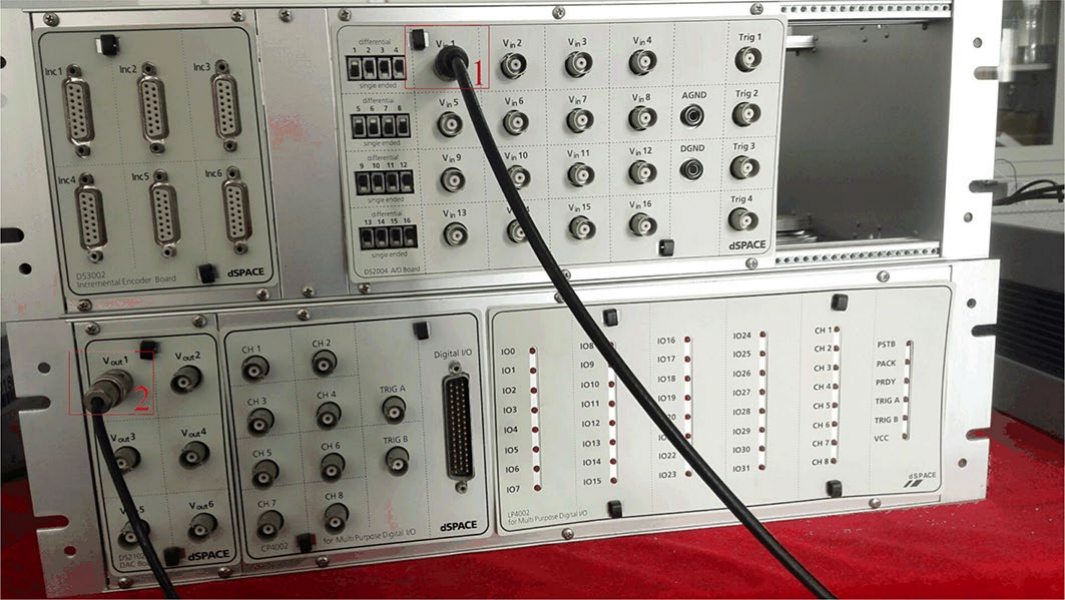
Connection board of dSPACE.

In this section, the experiments of modeling are all based on the dSPACE platform including D/A (DS2012) and A/D (DS2004) converters, which connect the dSPACE with the EMM. Figure 6 shows the connection board of dSPACE where plug 1 is used to send out the control signal to the EMM to drive the slow-scanning axis while plug 2 is used to receive the amplified voltage denoting the angle position of mirror plate measured by the PRS. In addition, the sampling frequency is 5 $kHz$.

### Building simplified model

By neglecting the effect of nonlinear factors such as hysteresis^15,16^, the simplified model to describe the deflection angle of mirror plate of the EMM, driven by the slow-scanning axis is established by estimating the parameters $\theta^{T} (k)$ by the modeling method as shown in Modeling of EMM.

In the experiment of modeling the simplified model, the slow-scanning axis of EMM is excited by the input voltage with the form: $u(k) = 2e^{{\frac{ - k}{{3500}}}} sin(2\pi \times 300 \times e^{{\frac{ - k}{{3500}}}} )$ (*V*). By using the measured input and output data of EMM as well as the modeling method provided by Modeling of EMM, it obtains the model order result, i.e., $\hat{m} = 2,$ while $\Delta V(3,N) = 0.308, \, (N = 13927)$ and $\Delta V(2,N) = 4.1104, \, (N = 13927)$. Thus, it leads to $\Delta V(3,N) \ll \Delta V(2,N), \, (N = 13927)$ and the model order is chosen as $m = 2.$

**Figure 7:**
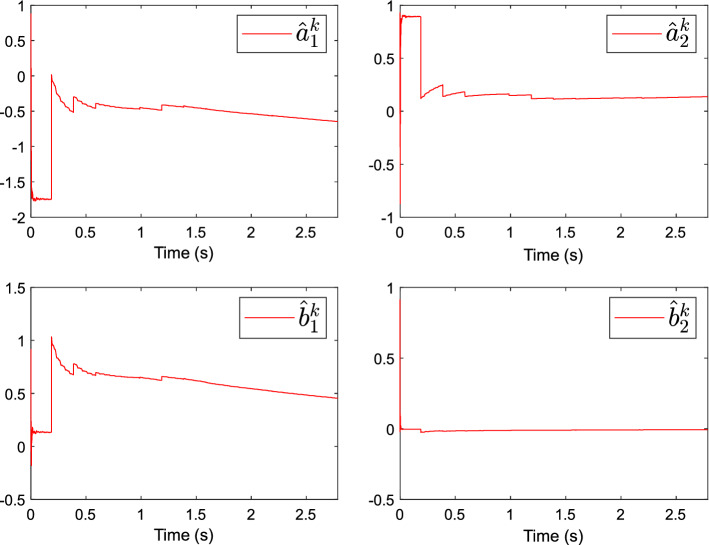
Convergence process of parameter estimation.

Then, the parameter convergence process of the simplified model using the RLS algorithm is shown in Fig. 7. The corresponding estimation of the model parameter vector is

$$ \theta^{T} (k) = [\begin{array}{*{20}c} {a_{s,1} } & {a_{s,2} } & {b_{s,1} } & {b_{s,2} } \\ \end{array} ] = [ - 0.6434, \, - 0.1371, \, 0.4563, - 0.0069]. $$

### Performance of model-guided extremum seeking scheme

Based on the estimated parameters, the corresponding state-space model is constructed, i.e.

$$ \begin{aligned} \left[ {\begin{array}{*{20}c} {x_{s,1}^{i} (k + 1)} \\ {x_{s,2}^{i} (k + 1)} \\ \end{array} } \right] & = \left[ {\begin{array}{*{20}c} 0 & 1 \\ { - 0.1371} & { - 0.6434} \\ \end{array} } \right]\left[ {\begin{array}{*{20}c} {x_{s,1}^{i} (k)} \\ {x_{s,2}^{i} (k)} \\ \end{array} } \right] + \left[ {\begin{array}{*{20}c} {0.4563} \\ {0.2936} \\ \end{array} } \right]u_{s}^{i} (k) \hfill \\ y_{s}^{i} (k) & = [\begin{array}{*{20}c} 1 & 0 \\ \end{array} ]\left[ {\begin{array}{*{20}c} {x_{s,1}^{i} (k)} \\ {x_{s,2}^{i} (k)} \\ \end{array} } \right] \hfill \\ \end{aligned} $$

where the physical meaning of $x_{s,1}^{i} (k)$ denotes the angle position of the mirror plate while $x_{s,2}^{i} (k)$ represents the velocity of angle.

**Figure 8:**
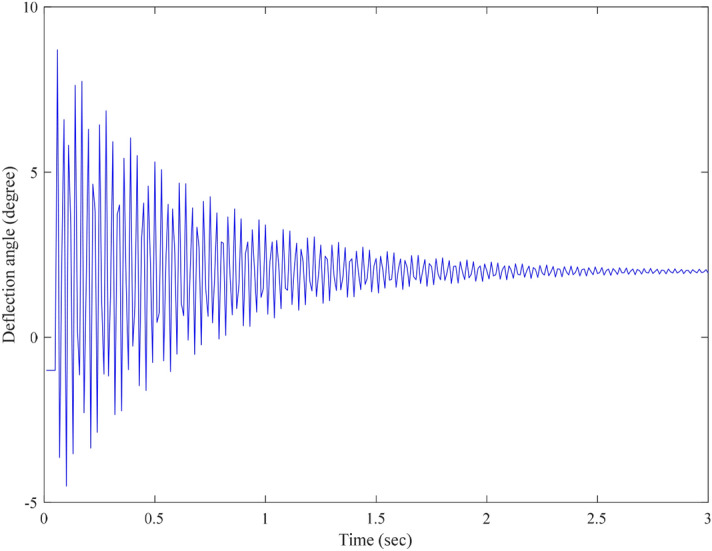
Open-loop step response of EMM disturbed by random noise.

**Figure 9:**
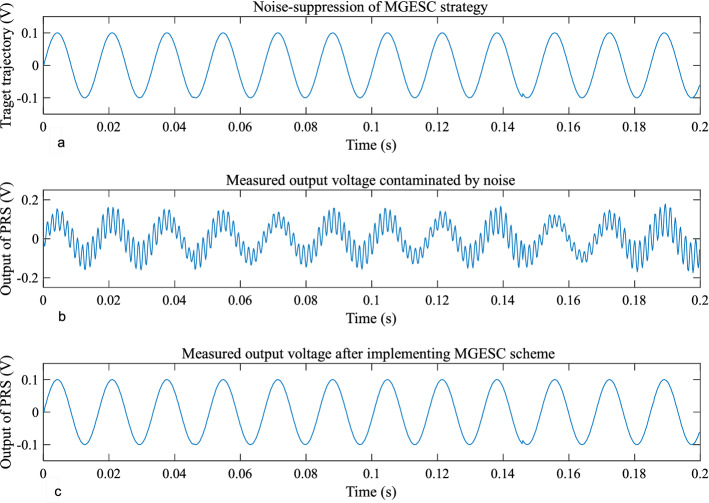
Noise suppression of the MGESC scheme in time-domain.

**Figure 10:**
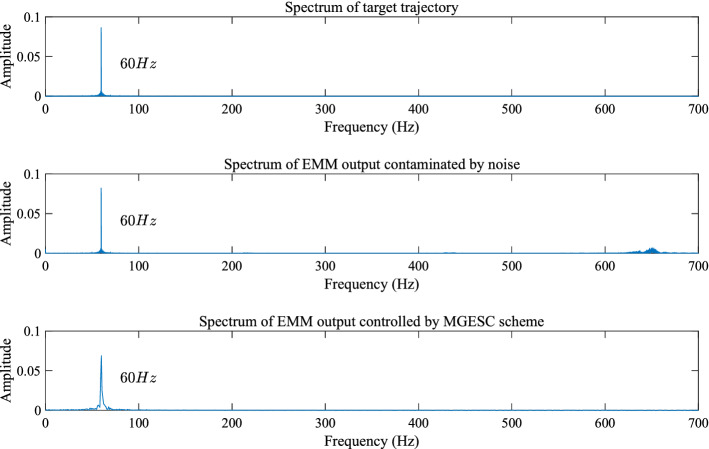
Spectrum analysis of the noise suppression using MGESC scheme.

**Figure 11:**
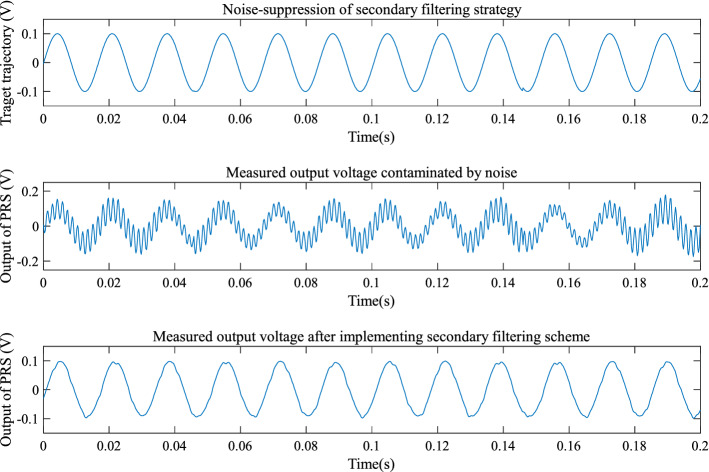
Noise suppression of the secondary filter in time-domain.

**Figure 12:**
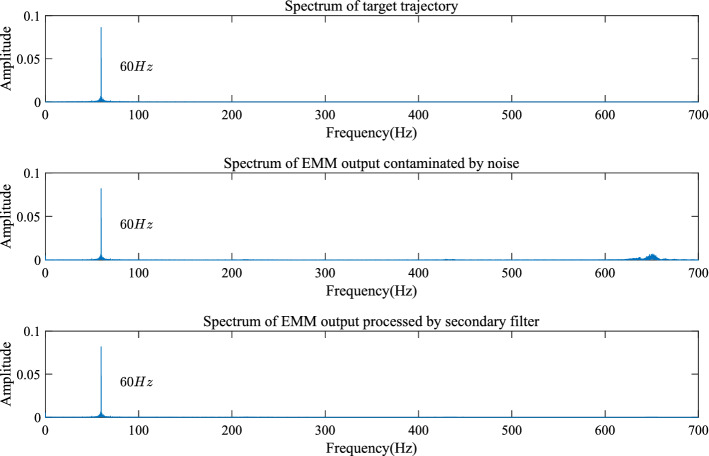
Spectrum analysis of the noise suppression using secondary filter.

From the experimental results, it is seen that the open-loop step response of the EMM shown in Fig. 8 is affected by random noise. This is because electromagnetic interference acts on the measurement signal of the piezoresistive sensor. It is also found out that the proposed MGESC strategy has demonstrated good characteristic of noise-suppression. In the noise suppression experiment, the EMM is placed close to the radio noise generator, which generates electromagnetic random noise with a mean value of zero and a variance of 0.025. Figure 9 shows that the noise suppression result of the proposed MGESC method in time-domain while Fig. 10 is the corresponding spectrum of the noise elimination performance of the MGESC scheme. It is seen that the high-frequency noise has been removed when the proposed MGESC scheme is applied to the EMM. For comparison, the secondary filtering approach developed in Ref.^8^ is also used to suppress the influence of noise on the output measurement of EMM. Figure 11 shows the corresponding filtering result of the secondary filter in time-domain. On the other hand, Fig. 12 illustrates the secondary filtering result of the output of EMM in frequency domain.

From Figs. 9, 10 and 12, it is seen that the capability of noise suppression of the proposed MGESC method is very similar to that of the secondary filter. However, the MGESC scheme does avoid the complicated design process of the secondary filter^8^. However, by comparing Figs. 9 with 11, it is known that the MGESC scheme has achieved smoother filtering result than that of the secondary filtering method.

#### *Remark 2*

 It can be seen from Figs. 10 and 12 that the MGESC strategy proposed in this paper has a noise suppression effect. This filtering effect is like the secondary filter. However, due to this noise suppression function, there is no need to specially design a secondary filter to filter out the interference of random noise to the system. This simplifies the design of the system, which is an interesting advantage of the MGESC method.

Subsequently, the simulation results on angle control of the EMM will be presented in the following.

In the simulation, the EMM is described by:

$$ y(k) = - 0.66y(k - 1) - 0.139y(k - 2) + 0.44u(k - 1) - 0.0072u(k - 2) + e(k) $$

where e(*k*) is supposed to a white noise sequence with zero-mean value and variance 0.15. It is also assumed that the simplified model is of the form, i.e.,

$$ \begin{aligned} \left[ {\begin{array}{*{20}c} {x_{s,1}^{i} (k + 1)} \\ {x_{s,2}^{i} (k + 1)} \\ \end{array} } \right] & = \left[ {\begin{array}{*{20}c} 0 & 1 \\ { - 0.1371} & { - 0.6434} \\ \end{array} } \right]\left[ {\begin{array}{*{20}c} {x_{s,1}^{i} (k)} \\ {x_{s,2}^{i} (k)} \\ \end{array} } \right] + \left[ {\begin{array}{*{20}c} {0.4563} \\ {0.2936} \\ \end{array} } \right]u_{s}^{i} (k) \hfill \\ y_{s}^{i} (k) & = [\begin{array}{*{20}c} 1 & 0 \\ \end{array} ]\left[ {\begin{array}{*{20}c} {x_{s,1}^{i} (k)} \\ {x_{s,2}^{i} (k)} \\ \end{array} } \right]. \hfill \\ \end{aligned} $$

It is seen that the model residual of parameters exists in the simplified model. Based on this setup, the corresponding simulation results will be presented in the following.

**Figure 13:**
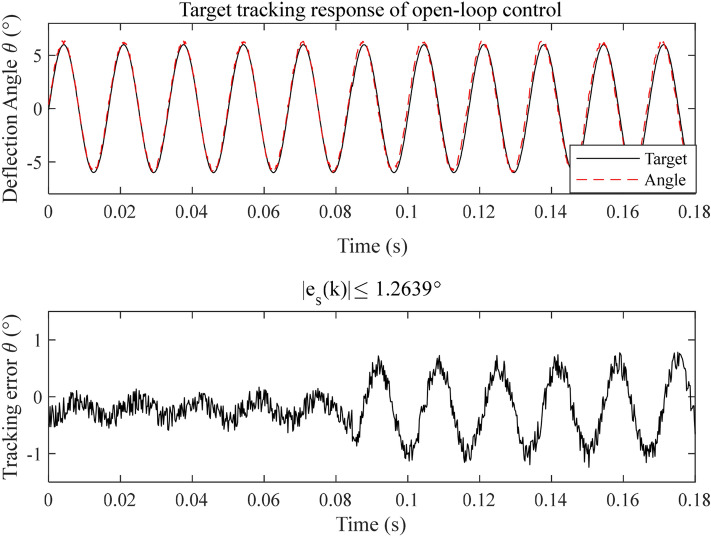
Tracking performance of the open-loop control.

**Figure 14:**
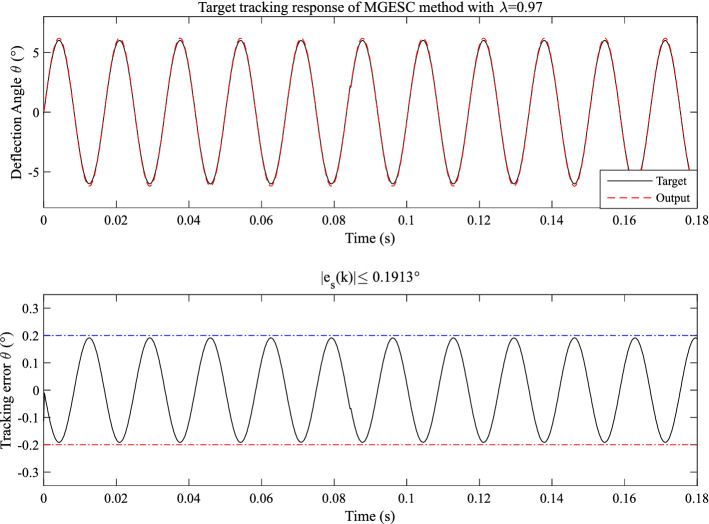
Tracking performance of the MGESC with fixed λ = 0.97.

First, the open-loop control scheme is applied to the EMM, the corresponding open-loop response is shown in Fig. 13. It is seen that the absolute tracking error bound of the system is 1.2639°. Moreover, the effect of noise can also obviously be seen in the tracking error. Apparently, it is not a satisfactory response. In order to reduce the tracking error, the proposed MGESC method is applied to the target trajectory tracking of the EMM. Figure 14 shows the control response of the MGESC strategy with a fixed step size of 0.97. The absolute tracking error obtained is less than 0.1913°, which is much smaller than the open loop control, but it is not acceptable for precision angle control of EMM.

**Figure 15:**
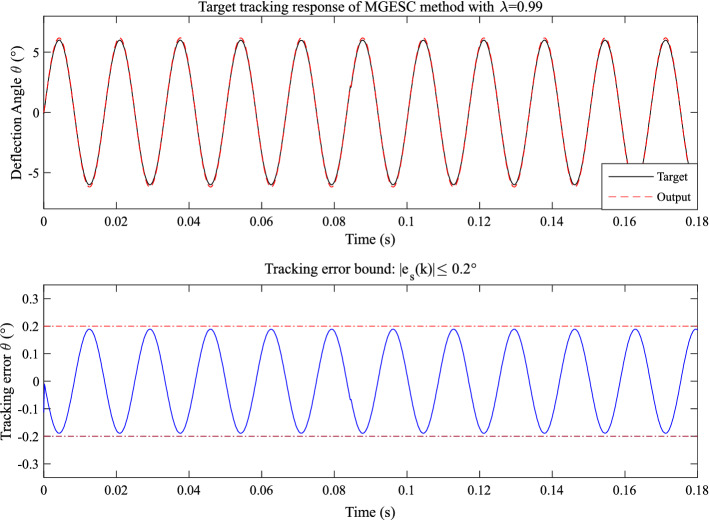
Tracking performance of the MGESC with fixed λ = 0.99.

**Figure 16:**
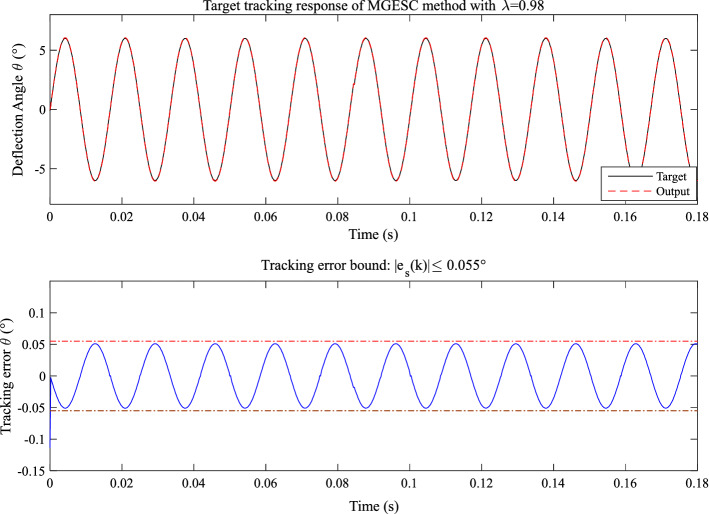
Tracking performance of the MGESC with fixed λ = 0.98.

In order to find a proper value of step-size, λ = 0.98 and λ = 0.99 are selected. The corresponding control response of the MGESC with λ = 0.99 and λ = 0.98 are shown in Figs. 15 and 16, respectively. It is seen that the absolute tracking error of the case that λ = 0.99 is within the bound of 0.2° while the absolute tracking error of the MGESC with λ = 0.98 is less than the error bound of 0.055°.

According to Figs. 14, 15 and 16, it is known that the value of optimization step-size has significant influence on the control performance of the MGESC strategy. The proposed MGESC method has shown its great potential in target tracking of the EMM. However, the selection of the step-size by empirical method is difficult and time-consuming, which cannot guarantee the best solution of control. Thus, seeking an optimal value of step-size will lead to the satisfactory control response of EMM.

**Figure 17:**
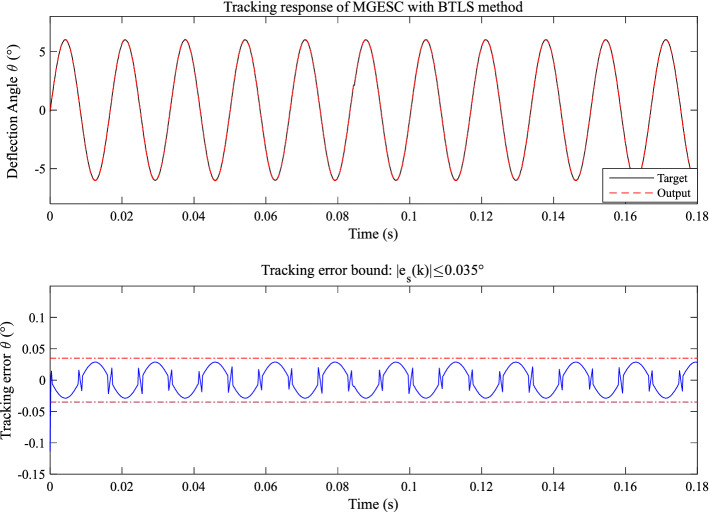
Tracking performance of the MGESC with BTLS method.

**Figure 18:**
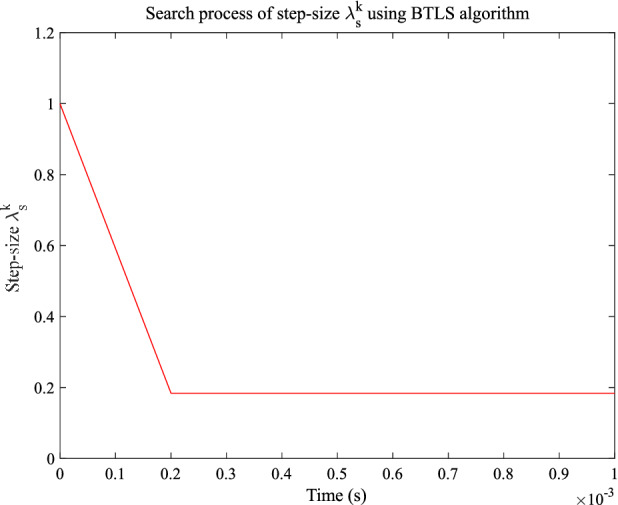
Search process of step-size using BTLS method.

In this paper, the proposed MGESC with BTLS method will provide us with the possibility to find the best value of step-size. Figure 17 shows the tracking control performance of the MGESC with BTLS method while Fig. 18 illustrates the search process of the step-size. Figure 17 shows that the initial value of the step-size has a significant impact on the system performance. From Fig. 17, it is seen that the initial absolute tracking error of the MGESC method is 0.13°. After t = 0.0002 s., the absolute tracking error of the MGESC is reduced to within the error band of 0.035°. Figure 18 illustrates that when the initial value of the step-size is 1, it results in a larger initial absolute value error. When t = 0.0002 s., the step-size converges to the optimal value of 0.2, the corresponding absolute value of the tracking error quickly enters the error band of 0.035°.

By comparing with cases where λ = 0.97, 0.98 and 0.99, the proposed MGESC with BTLS scheme has achieved the best tracking result. In addition, Fig. 18 also shows that the step-size search converges very fast to the optimal value, which leads to the satisfactory tracking response of EMM.

Moreover, by comparing with the case of open-loop control shown in Figs. 13, 14, 15, 16 and 17 have shown that the effect of random noise acting on the system has been removed by the proposed MGESC method. Thus, the noise suppression ability of the MGESC has been proved again by the simulation results.

## Conclusion

In this paper, the model-guided extremum seeking control scheme is proposed for the angle control by controlling the slow-scanning axis of electromagnetic micromirrors. Different from the other ESC strategies, the proposed method uses the information provided by the developed simplified model of the EMM as the start value to search for the best to improve the control performance of the system. The convergence of the MGESC scheme is also proved. In order to further improve the control performance, the backtracking line search strategy of step-size is presented. Simulation results show that the optimization step-size has significant influence on the tracking performance of the EMM system. By comparing with the open-loop control, and the MGESC method with fixed step-size, the proposed MGESC with BTLS method has obtained promising control response.

It is noted that even though the driving principle and mechanism of electromagnetic micromirrors are different from other types of MEMS micro-mirrors, such as electrostatic, electrothermal and piezoelectric actuated MEMS micro-mirror, the control scheme may still be used to control these micro-mirrors suppose the simplified model of these micro-mirrors are available. Moreover, the fast-scanning axis of the micro-mirror usually works around the resonant frequency about 20 kHz, which is controlled by a frequency lock controller. It does not seem to require the use of the control strategy, which is the same as that used in the slow scanning axis.

Moreover, the proposed control scheme also shows the capability to suppress the noise of the system, which is an interesting phenomenon worth to study further in the future.

## Supplementary Information

Supplementary Information.
