# Supplementary material for: Model guided extremum seeking control of electromagnetic micromirrors
Source: Sci Rep. 2021 Sep 2;11:17613. doi: 10.1038/s41598-021-97098-6 (PMC8413333; doi:10.1038/s41598-021-97098-6)
Supplement: Supplementary file 1 — Supplementary Information. [file 41598_2021_97098_MOESM1_ESM.docx]

Appendix A: The proof of convergence of MGESC with BTLS method

Set the cost function (26) as

$\Gamma(\bullet)=\Gamma(u_{s}^{i}(k))=J_{s}^{i}(k+1).$ (A.1)

According to Taylor’s Theorem [12], and along with (27) and (28) , it is obtained that

$\begin{matrix} \Gamma(u_{s}^{i}(k)+\Delta u_{s}^{i}(k)=\Gamma(u_{s}^{i}(k)-\lambda_{s}^{i}\nabla J_{s}^{i}(k+1)) \\ \text{ }=\Gamma(u_{s}^{i}(k))-\lambda_{s}^{i}\nabla J_{s}^{i}(k+1)\nabla\Gamma(u_{s}^{i}(k)))+o(\left\|-\lambda_{s}^{i}\nabla J_{s}^{i}(k+1)\right\|) \\ \text{ }=\Gamma(u_{s}^{i}(k))-\lambda_{s}^{i}(\nabla J_{s}^{i}(k+1))^{2}+o(\left\|-\lambda_{s}^{i}\nabla J_{s}^{i}(k+1)\right\|) \end{matrix}$ (A.2)

where $o(\left\|-\lambda_{s}^{i}\nabla J_{s}^{i}(k+1)\right\|)$ is infinitesimal value of$\left\|-\lambda_{s}^{i}\nabla J_{s}^{i}(k+1)\right\|$ . So, the term$-\lambda_{s}^{i}(\nabla J_{s}^{i}(k+1))^{2}$dominates in (A.2) for small$\lambda_{s}^{k}$and determined $\nabla J_{s}^{i}(k+1)$(see eq. (29)). There is positive but sufficiently small $\lambda_{s}^{k}$ (see Sec. III-B) such that

$-\lambda_{s}^{k}(\nabla J_{s}^{i}(k+1))^{2}<0.$ (A.3)

Considering (A.2), it yields

$\Gamma(u_{s}^{i}(k)+\Delta u_{s}^{i}(k))<\Gamma(u_{s}^{i}(k)).$ (A.4)

Suppose $J_{s}^{i}(k+1)$is continuously differentiable in an open neighborhood of the local minimum solution$u_{s}^{*}(k)$. Let

$\nabla J_{s}^{i}(k+1)=0$ (A.5)

and along with (29) it derives

$r_{s}(k+1)-y_{s}^{i}(k+1)=0$ (A.6)

and

$u_{s}^{i}(k)=u_{s}^{*}(k).$ (A.7)

When the iteration stop criterion shown in (31) is satisfied, then

$J_{s}^{i}(k+1)\to0$, (A.8)

$y_{s}^{i}(k+1)\to r_{s}(k+1)$ , (A.9)

and

$u_{s}^{i}(k)\to u_{s}^{*}(k)$. (A.10)

Thus, this new MGESC with BTLS algorithm is derived.
